# Integrated analysis of the functions and prognostic values of RNA-binding proteins in neuroblastoma

**DOI:** 10.1371/journal.pone.0260876

**Published:** 2021-12-08

**Authors:** Jun Yang, Jiaying Zhou, Cuili Li, Shaohua Wang

**Affiliations:** 1 Department of Pediatrics, The University of Hong Kong-ShenZhen Hospital, ShenZhen, China; 2 Department of Pediatrics, Women and Children Institute of Futian, University of South China, ShenZhen, China; University of Science and Technology Liaoning, CHINA

## Abstract

**Background:**

Neuroblastoma (NB) is the most common solid tumor in children. NB treatment has made significant progress; however, given the high degree of heterogeneity, basic research findings and their clinical application to NB still face challenges. Herein, we identify novel prognostic models for NB.

**Methods:**

We obtained RNA expression data of NB and normal nervous tissue from TARGET and GTEx databases and determined the differential expression patterns of RNA binding protein (RBP) genes between normal and cancerous tissues. Lasso regression and Cox regression analyses identified the five most important differentially expressed genes and were used to construct a new prognostic model. The function and prognostic value of these RBPs were systematically studied and the predictive accuracy verified in an independent dataset.

**Results:**

In total, 348 differentially expressed RBPs were identified. Of these, 166 were up-regulated and 182 down-regulated RBPs. Two hubs RBPs (*CPEB3* and *CTU1*) were identified as prognostic-related genes and were chosen to build the prognostic risk score models. Multivariate Cox analysis was performed on genes from univariate Cox regression and Lasso regression analysis using proportional hazards regression model. A five gene prognostic model: Risk score = (-0.60901*expCPEB3)+(0.851637*expCTU1) was built. Based on this model, the overall survival of patients in the high-risk subgroup was lower (P = 2.152e-04). The area under the curve (AUC) of the receiver-operator characteristic curve of the prognostic model was 0.720 in the TARGET cohort. There were significant differences in the survival rate of patients in the high and low-risk subgroups in the validation data set GSE85047 (P = 0.1237e-08), with the AUC 0.730. The risk model was also regarded as an independent predictor of prognosis (HR = 1.535, 95% CI = 1.368–1.722, P = 2.69E-13).

**Conclusions:**

This study identified a potential risk model for prognosis in NB using Cox regression analysis. RNA binding proteins (*CPEB3* and *CTU1*) can be used as molecular markers of NB.

## Introduction

Neuroblastoma (NB) is the main cause of tumor-related deaths in children worldwide [1]. Diagnosis and treatment have made great progress in the past 20 years and the average 5-year relative survival rate of NB has reached 50% [2]. Currently, the diagnosis of NB mainly relies on histopathological examination, imaging results, and molecular biomarkers [3]. Early detection of NB is difficult. This may be the most important factor affecting the mortality of patients with NB [4]. Therefore, further study of the molecular mechanisms underlying NB and identification of effective molecular markers for early cancer screening are essential to enhance the therapeutic outcomes and quality of life of children [5].

RNA binding proteins (RBPs) are pleiotropic proteins that can regulate gene expression by interacting with various types of RNA [6], including rRNA, ncRNA, snRNA, miRNA, mRNA, tRNA, and snoRNA [7]. Currently, over 1500 different types of RBPs have been identified in the human genome through whole-genome sequencing [8]. Specifically the ribonucleoprotein complex formed by the binding of RBP and target RNA regulates the stability of mRNA at the post-transcriptional level, thereby affecting RNA processing, splicing, localization, export, and translation [9–11]. Ultimately, post-transcriptional modification of mRNA leads to the regulation of various important physiological processes of cells [12]. Current research has found that RBPs play an important role in many human diseases and are key regulators of the development and progression of cardiovascular diseases [13], myotonic muscular dystrophy [14], neurological diseases [15], and cancer [16].

In recent years, new bioinformatics approaches, such as bipartite network projection, IRWNRLPI (Integrating Random Walk and Neighborhood Regularized Logistic Matrix Factorization for lncRNA-Protein Interaction), and HLPI-Ensemble, have greatly improved the research and prediction of RBP function [17–19]. Herein, we used high-throughput bioinformatics analysis to identify RBPs that are differentially expressed in cancer samples and normal tissue samples, and systematically investigated their expression profiles, functional effects, and potential mechanisms to understand their role in tumors. This study will deepen our understanding of the molecular mechanism of NB and provide potential diagnostic or prognostic biomarkers for NB.

## Materials and methods

### Data sets and preprocessing

We obtained RNA expression datasets and corresponding clinical data of NB patients from the Therapeutically Applicable Research To Generate Effective Treatments project database (TARGET, https://target-data.nci.nih.gov/Public/NBL/mRNA-seq/L3/), and normal neural tissues samples datasets from Genotype-Tissue Expression Database (GTEx, https://gtexportal.org/), respectively. All data derived from an open access data platform, and thus, this study did not require ethics committee approval. To determine differentially expressed genes between NB tissue and normal samples, the Limma software package was used for analysis. The GSE85047 dataset was downloaded from Gene Expression Omnibus (GEO) (https://www.ncbi.nlm.nih.gov/geo/query/acc.cgi?acc=gse85047) and was used as a validation cohort.

### Gene ontology enrichment and KEGG pathway analysis

Gene enrichment analysis and pathway analysis was carried out using the R package “clusterProfiler” [20].

### Protein-–protein interaction network building and subnet detection

Differential protein–protein interaction (PPI) information for RBP was evaluated using the STRING database (http://www.string-db.org/) [21] and further building and visualization of the PPI network was performed using Cytoscape 3.7.0 software. We used the molecular complexity detection (MCODE) plug-in to cluster genes in the PPI network and to build functional modules. A P-value<0.05 was considered a statistically significant difference [22].

### Prognostic model construction

The R package “survival” was applied to carry out univariate Cox regression analysis using all differentially expressed RBPs to identify prognostic genes, and Lasso regression was performed to further screen important key genes. Finally, based on the preliminary screening of the above key candidate genes, we built a multivariate Cox proportional hazard regression model and evaluated the survival of patients using the following risk score formula:

Riskscore=β1*Exp1+β2Exp2+…+βiExpi (1)

where, β was the value of the risk coefficient, and Exp represented the value of the expression of a certain gene. Based on the median value of the risk score, NB patients were divided into two groups: low-risk group and high-risk group, and the survival differences between the two subgroups were compared through survival analysis. In addition, the prognostic ability of the above model was estimated through receiver operating characteristic curve (ROC) analysis. A sample of 276 NB patients with reliable follow-up information from the GSE85047 data set was used as the validation group to evaluate the predictive power of the prognostic model. P<0.05 was considered a statistically significant difference.

#### Constructing the lncRNA-miRNA-mRNA network of key RBPs

To study the relationship of lncRNA, miRNA and mRNA, based on the interactions of miRNA-lncRNA and miRNA-mRNA, the online database such as starBase (http://starbase.sysu.edu.cn/agoClipRNA.php?), targetscan(http://www.targetscan.org/vert_72/) [23], and LncBase (http://carolina.imis.athenannovation.gr/diana_tools/web/index.php?r=lncbasev2%2Findex) were used to build the lncRNA-miRNA-mRNA network.

## Results

### Identification of differently expressed RBPs in NB patients

**Fig 1:**
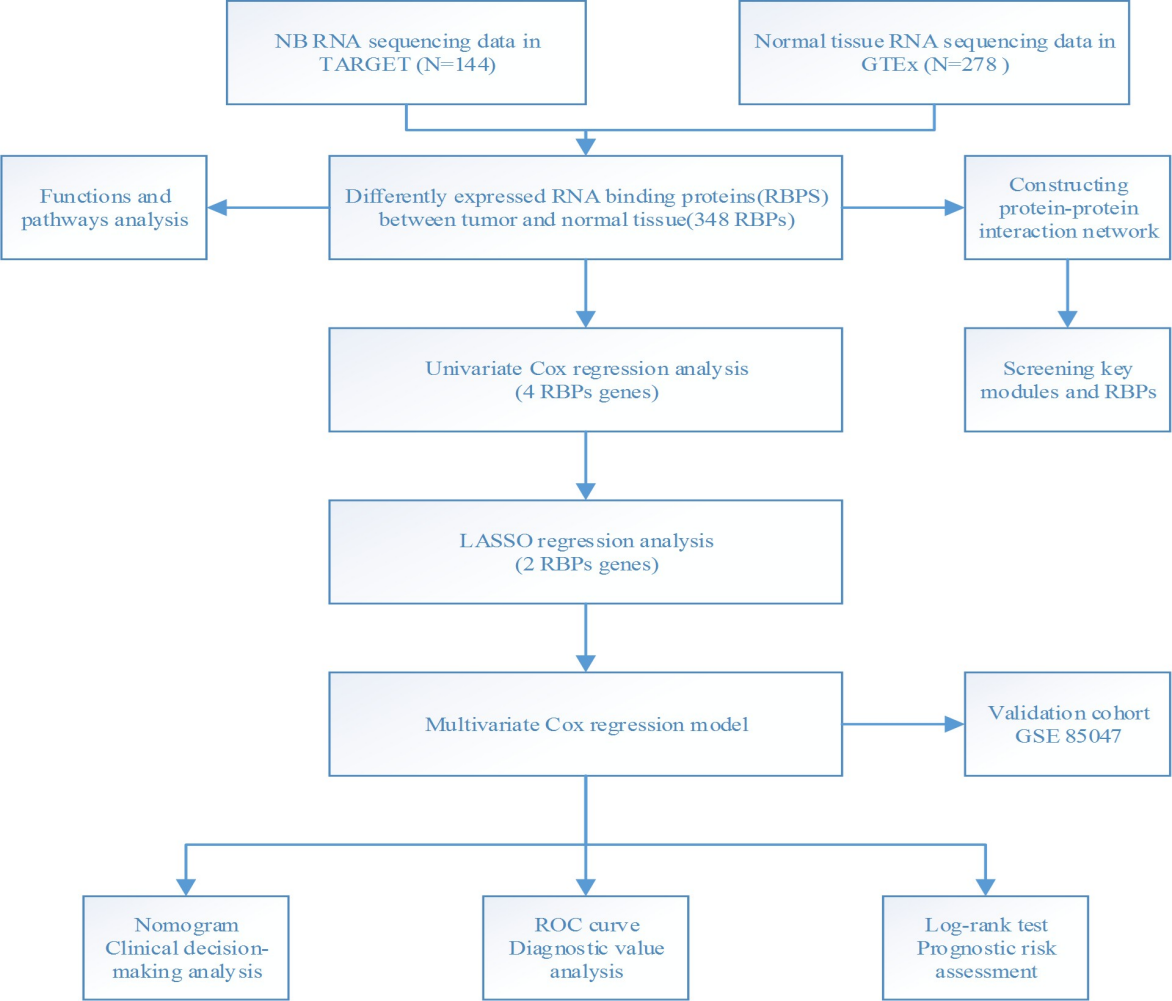
Framework for analyzing the RBPs in neuroblastoma.

**Fig 2:**
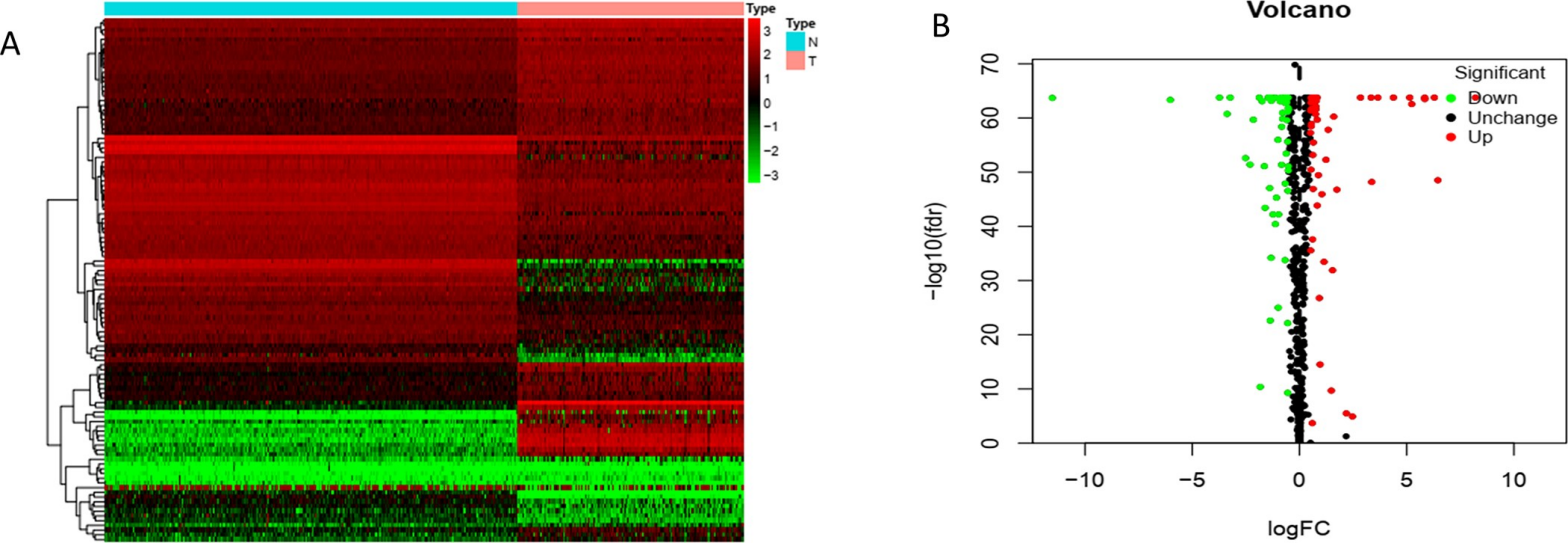
The differentially expressed RBPs in NB and GTEx samples. (A) Heat map; (B) Volcano plot.

We performed a methodical analysis of the key role and prognostic value of RBP in NB. The study workflow is illustrated in Fig 1. The NB data was downloaded from TARGET, which contained 144 tumor samples, and the normal nerve tissue data was downloaded from the GTEx database and contained 278 samples. After analyzing the currently known 1542 RBPs, 348 RBPs with significant differences in expression (P-adjusted <0.05, |log_2_ fold change [FC]|>1.0) were identified, and comprised 166 up-regulated RBPs and 182 down-regulated RBPs (Fig 2) (S1 Table).

### Enrichment analysis of the differently expressed RBPs

**Fig 3:**
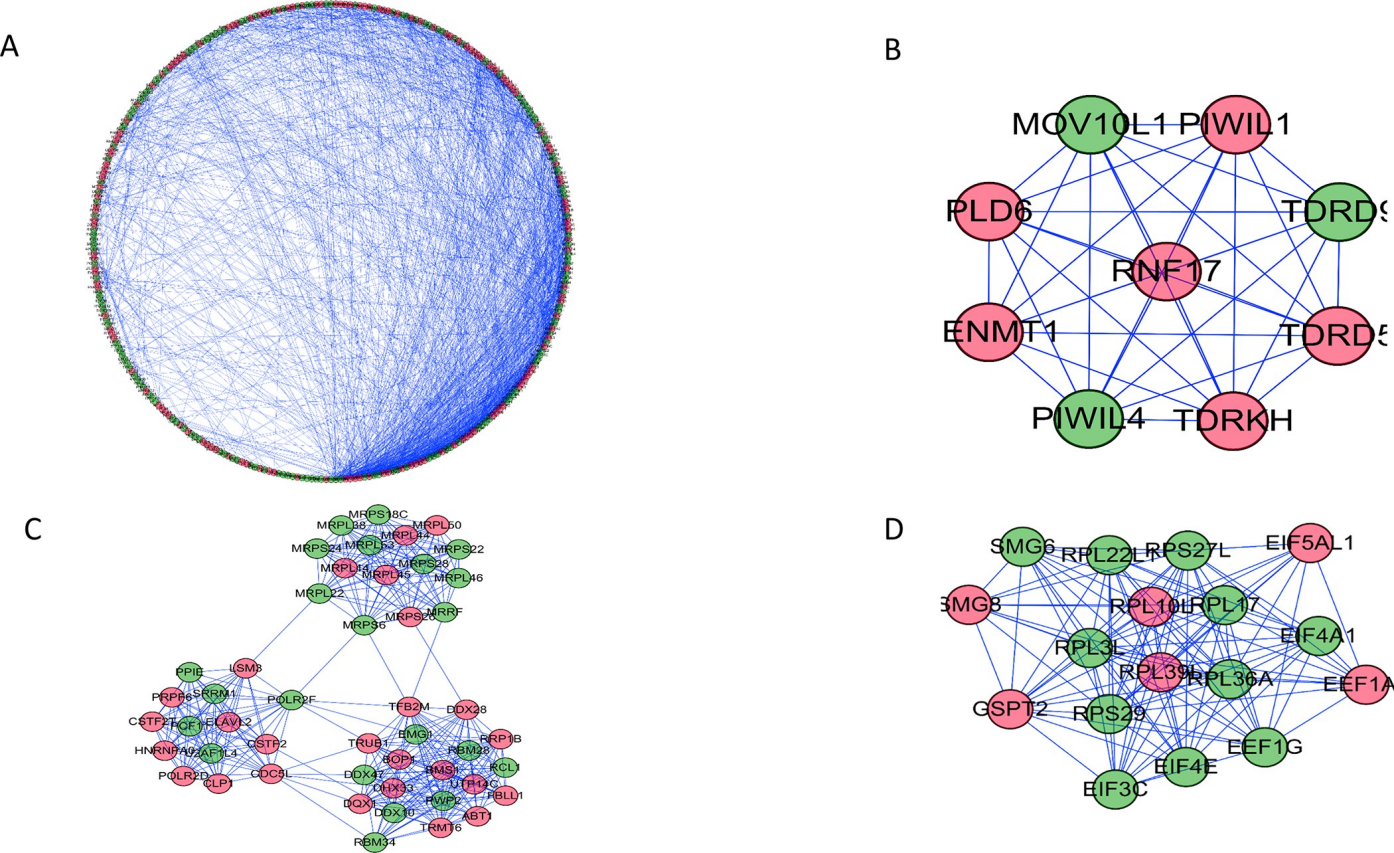
PPI network and subnet analysis. (A) Protein–protein interaction network of RBPs; (B) critical module 1 from PPI network; (C) critical module 2 from PPI network. (D) critical module 3 from PPI network. green: down-regulation with a fold change of more than 2; red: up-regulation with fold change of more than 2.

In order to study the functions and mechanisms of the selected RBP, we used the R package clusterProfiler for enrichment analysis. The results showed that enriched biological processes mainly involved mRNA processing, RNA splicing; ncRNA metabolic processes; RNA phosphodiester bond hydrolysis; RNA splicing, via transesterification reactions with bulged adenosine as a nucleophile; mRNA splicing, via spliceosome; RNA splicing, via transesterification reactions; nucleic acid phosphodiester bond hydrolysis; and RNA catabolic processes. Molecular functions included catalytic activity, acting on RNA ribonuclease activity; nuclease activity; mRNA 3’-UTR binding; endonuclease activity; translation regulator activity; catalytic activity, acting on a tRNA; mRNA binding; double-stranded RNA binding; endoribonuclease activity; and single-stranded RNA binding. Cellular components involved mainly the ribonucleoprotein granule, cytoplasmic ribonucleoprotein granule, ribosome, ribosomal subunit, organellar ribosome, mitochondrial ribosome, P-body, mitochondrial matrix, P granule, and pole plasm. The KEGG analysis [24] indicated mainly enrichment in RNA transport, the mRNA surveillance pathway, ribosome biogenesis in eukaryotes, RNA degradation, ribosome, aminoacyl-tRNA biosynthesis, spliceosome, and the RNA polymerase, Influenza A pathway (Fig 3) (Tables 1 and 2).

**Table 1:** Results of GO enrichment.

ONTOLOGY	ID	Description	pvalue	geneID	Count
BP	GO:0006397	mRNA processing	2.30E-47	CDC5L/RBFOX1/RBFOX2/RBFOX3/SRSF12/ELAVL2/ELAVL4/NCBP2L/RBM28/SREK1/APOBEC2/APOBEC1/RRP1B/CLP1/ZMAT5/MBNL1/ADARB2/LSM3/PRPF6/AFF2/A1CF/RNF113A/U2AF1/U2AF1L4/SNUPN/GEMIN6/RBMY1B/CSDC2/RNPC3/ZFP36L1/POLR2D/KHDRBS2/KHDRBS3/QKI/SRRM1/CSTF2/CSTF2T/RBM24/LSM11/HNRNPA0/HNRNPA1L2/DDX47/PCF11/FASTKD5/SMN1/SMN2/RBM11/TSEN2/POLR2F/ZNF473/CELF3/CELF4/CELF5/CELF6/CPEB1/CPEB3/RPUSD3/RBM15B/RBM15/SNIP1/PPIE/PRPF40B/HTATSF1/ESRP1/ESRP2/ARL6IP4/RNGTT/NOVA1/NOVA2/SARNP/SRRM4/RBM4	72
BP	GO:0008380	RNA splicing	9.69E-37	CDC5L/RBFOX1/RBFOX2/RBFOX3/SRSF12/ELAVL2/NCBP2L/RBM28/SREK1/RRP1B/CLP1/ZMAT5/MBNL1/CLK3/CLK4/LSM3/PRPF6/AFF2/RNF113A/U2AF1/U2AF1L4/SNUPN/GEMIN6/RBMY1B/RNPC3/POLR2D/KHDRBS2/KHDRBS3/QKI/SRRM1/CSTF2/CSTF2T/RBM24/HNRNPA0/HNRNPA1L2/DDX47/PCF11/SMN1/SMN2/RBM11/TSEN2/POLR2F/CELF3/CELF4/CELF5/CELF6/RBM15B/RBM15/SNIP1/PPIE/PRPF40B/HTATSF1/ESRP1/ESRP2/ARL6IP4/NOVA1/NOVA2/SRRM4/RBM4	59
BP	GO:0034660	ncRNA metabolic process	4.35E-34	METTL2A/TDRD9/TRMT5/RPP25/DARS2/HENMT1/TRMT10C/RCL1/ADAT2/BOP1/EXOSC7/PWP2/CLP1/PLD6/EXOSC6/INTS8/EMG1/TDRKH/TRMT6/SMAD2/SMAD3/PSTK/MOV10L1/PIWIL1/PIWIL4/POP7/THG1L/BMS1/SARS2/ANG/POP4/RC3H1/AARSD1/POLR2D/INTS3/CSTF2/INTS7/AARS2/UTP14C/DDX47/NPM3/PUS3/TSEN2/POLR2F/TRDMT1/CELF3/FARSB/ISG20/ZC3H8/POP1/INTS2/BCDIN3D/NAF1/CTU1/INTS6/LIN28A/LIN28B/MARS2	58
BP	GO:0090501	RNA phosphodiester bond hydrolysis	5.45E-34	REXO2/RPP25/RNASEK/PNLDC1/RCL1/BOP1/EXOSC6/AZGP1/RNASE10/MOV10/PIWIL1/PIWIL4/POP7/RNASE2/RNASE3/RNASE4/RNASE7/RNASE8/ANG/POP4/CNOT2/MRPL44/RNASE11/ENDOU/ERI2/RNASE13/SAMHD1/PCF11/TSEN2/ISG20/POP1/RNASEH2A/CNOT6/SMG6/RNASET2/EXO1	36
BP	GO:0000377	RNA splicing, via transesterification reactions with bulged adenosine as nucleophile	1.62E-26	CDC5L/RBFOX2/SRSF12/ELAVL2/NCBP2L/SREK1/CLP1/ZMAT5/LSM3/PRPF6/RNF113A/U2AF1/U2AF1L4/SNUPN/GEMIN6/RNPC3/POLR2D/KHDRBS2/KHDRBS3/SRRM1/CSTF2/CSTF2T/RBM24/HNRNPA0/PCF11/SMN1/SMN2/RBM11/POLR2F/CELF3/CELF4/CELF5/CELF6/RBM15B/RBM15/SNIP1/PPIE/PRPF40B/HTATSF1/ESRP2/NOVA1/NOVA2/SRRM4/RBM4	44
BP	GO:0000398	mRNA splicing, via spliceosome	1.62E-26	CDC5L/RBFOX2/SRSF12/ELAVL2/NCBP2L/SREK1/CLP1/ZMAT5/LSM3/PRPF6/RNF113A/U2AF1/U2AF1L4/SNUPN/GEMIN6/RNPC3/POLR2D/KHDRBS2/KHDRBS3/SRRM1/CSTF2/CSTF2T/RBM24/HNRNPA0/PCF11/SMN1/SMN2/RBM11/POLR2F/CELF3/CELF4/CELF5/CELF6/RBM15B/RBM15/SNIP1/PPIE/PRPF40B/HTATSF1/ESRP2/NOVA1/NOVA2/SRRM4/RBM4	44
BP	GO:0000375	RNA splicing, via transesterification reactions	2.64E-26	CDC5L/RBFOX2/SRSF12/ELAVL2/NCBP2L/SREK1/CLP1/ZMAT5/LSM3/PRPF6/RNF113A/U2AF1/U2AF1L4/SNUPN/GEMIN6/RNPC3/POLR2D/KHDRBS2/KHDRBS3/SRRM1/CSTF2/CSTF2T/RBM24/HNRNPA0/PCF11/SMN1/SMN2/RBM11/POLR2F/CELF3/CELF4/CELF5/CELF6/RBM15B/RBM15/SNIP1/PPIE/PRPF40B/HTATSF1/ESRP2/NOVA1/NOVA2/SRRM4/RBM4	44
BP	GO:0090305	nucleic acid phosphodiester bond hydrolysis	1.28E-24	REXO2/ZC3H12C/RPP25/RNASEK/PNLDC1/RCL1/BOP1/PLD6/EXOSC6/AZGP1/RNASE10/MOV10/PIWIL1/PIWIL4/POP7/RNASE2/RNASE3/RNASE4/RNASE7/RNASE8/ANG/POP4/CNOT2/MRPL44/RNASE11/ENDOU/ERI2/RNASE13/SAMHD1/PCF11/TSEN2/ISG20/POP1/RNASEH2A/CNOT6/SMG6/RNASET2/EXO1	38
BP	GO:0006401	RNA catabolic process	2.55E-24	PSMA6/TNPO1/GSPT2/PNLDC1/EXOSC7/APOBEC1/EXOSC6/OAS2/LSM3/IGF2BP1/IGF2BP2/IGF2BP3/SMG8/MOV10/RBM46/RPS29/SIDT2/RPL17/RNASE2/RNASE3/RC3H1/ZFP36L1/CNOT2/RBM24/HNRNPA0/CPEB3/MEX3D/ISG20/POP1/YBX3/RNASEH2A/RNH1/CNOT6/SMG6/NAF1/RNASET2/RPL36A/WDR61/NANOS1/NANOS2/NANOS3/LIN28A/LIN28B	43
BP	GO:0034470	ncRNA processing	3.39E-23	METTL2A/TRMT5/RPP25/TRMT10C/RCL1/ADAT2/BOP1/EXOSC7/PWP2/CLP1/EXOSC6/INTS8/EMG1/TRMT6/SMAD2/SMAD3/POP7/THG1L/BMS1/POP4/INTS3/CSTF2/INTS7/UTP14C/DDX47/NPM3/PUS3/TSEN2/TRDMT1/ISG20/POP1/INTS2/BCDIN3D/NAF1/CTU1/INTS6/LIN28A/LIN28B	38
CC	GO:0035770	ribonucleoprotein granule	7.92E-24	PSMA6/TDRD9/HENMT1/APOBEC3G/TDRD5/TDRKH/MBNL1/IGF2BP1/MOV10/MOV10L1/PIWIL1/PIWIL4/DDX25/RC3H1/ZFP36L1/ATXN2/RBPMS/DDX28/FASTKD5/SMN1/SMN2/CPEB1/MEX3A/MEX3B/EIF4E/NANOS2/NANOS3/SARNP/LIN28A/RBM4/TOP1/JAKMIP1	32
CC	GO:0036464	cytoplasmic ribonucleoprotein granule	2.85E-21	PSMA6/TDRD9/HENMT1/APOBEC3G/TDRD5/TDRKH/MBNL1/IGF2BP1/MOV10/MOV10L1/PIWIL1/PIWIL4/DDX25/RC3H1/ZFP36L1/ATXN2/RBPMS/SMN1/SMN2/CPEB1/MEX3A/MEX3B/EIF4E/NANOS2/NANOS3/SARNP/LIN28A/RBM4/TOP1	29
CC	GO:0005840	ribosome	5.16E-15	MRPL53/EIF2D/MRPL38/MRPS24/MTG1/MRPS28/RPL10L/RPS29/MRPS22/RPL17/MRPL50/MRPS26/MRPS18C/MRPL45/NUFIP1/MRPL44/EIF2AK2/EIF2AK4/MRPL22/RPL3L/MRPL46/MRPL14/RPL39L/NR0B1/RPL36A/MRPS6/MRPL23	27
CC	GO:0044391	ribosomal subunit	5.48E-14	MRPL53/EIF2D/MRPL38/MRPS24/MRPS28/RPL10L/RPS29/MRPS22/RPL17/MRPL50/MRPS26/MRPS18C/MRPL45/MRPL44/MRPL22/RPL3L/MRPL46/MRPL14/RPL39L/RPL36A/MRPS6/MRPL23	22
CC	GO:0000313	organellar ribosome	2.70E-13	MRPL53/MRPL38/MRPS24/MTG1/MRPS28/MRPS22/MRPL50/MRPS26/MRPS18C/MRPL45/MRPL44/MRPL22/MRPL46/MRPL14/MRPS6/MRPL23	16
CC	GO:0005761	mitochondrial ribosome	2.70E-13	MRPL53/MRPL38/MRPS24/MTG1/MRPS28/MRPS22/MRPL50/MRPS26/MRPS18C/MRPL45/MRPL44/MRPL22/MRPL46/MRPL14/MRPS6/MRPL23	16
CC	GO:0000932	P-body	9.05E-13	PSMA6/APOBEC3G/MOV10/RC3H1/ZFP36L1/RBPMS/CPEB1/MEX3A/MEX3B/EIF4E/NANOS2/NANOS3/LIN28A/TOP1	14
CC	GO:0005759	mitochondrial matrix	6.67E-12	REXO2/MRPL53/TRMT5/DARS2/TRMT10C/MRPL38/MRPS24/MTG1/MRPS28/MRRF/MRPS22/SARS2/MRPL50/MRPS26/MRPS18C/MRPL45/PARS2/MRPL44/MRPL22/DDX28/TFB2M/FASTKD5/MRPL46/CARS2/MRPL14/RPUSD3/TERT/MRPS6/TST/MRPL23/MARS2	31
CC	GO:0043186	P granule	7.47E-10	TDRD9/HENMT1/TDRD5/TDRKH/MOV10L1/PIWIL1/PIWIL4	7
CC	GO:0045495	pole plasm	7.47E-10	TDRD9/HENMT1/TDRD5/TDRKH/MOV10L1/PIWIL1/PIWIL4	7
MF	GO:0140098	catalytic activity, acting on RNA	8.68E-30	METTL2A/TRMT5/RPP25/DARS2/HENMT1/TRMT10C/RNASEK/PNLDC1/EXOSC7/AZGP1/EMG1/EIF4A1/MOV10/MOV10L1/PIWIL1/POP7/SARS2/RNASE2/RNASE4/RNASE7/RNASE8/ANG/POP4/DDX25/PUS10/CNOT2/AARSD1/POLR2D/AARS2/ENDOU/SAMHD1/PUS3/TRDMT1/FARSB/TERT/ISG20/POP1/DDX10/BCDIN3D/RNASEH2A/CNOT6/SMG6/RNASET2/EXO1/MARS2	45
MF	GO:0004540	ribonuclease activity	1.45E-19	RPP25/RNASEK/PNLDC1/EXOSC7/AZGP1/PIWIL1/POP7/RNASE2/RNASE4/RNASE7/RNASE8/ANG/POP4/CNOT2/ENDOU/SAMHD1/ISG20/POP1/RNASEH2A/CNOT6/SMG6/RNASET2/EXO1	23
MF	GO:0004518	nuclease activity	1.49E-18	REXO2/ZC3H12C/RPP25/RNASEK/PNLDC1/EXOSC7/PLD6/AZGP1/PIWIL1/POP7/RNASE2/RNASE3/RNASE4/RNASE7/RNASE8/ANG/POP4/CNOT2/RNASE11/ENDOU/SAMHD1/ISG20/POP1/RNASEH2A/CNOT6/SMG6/RNASET2/EXO1	28
MF	GO:0003730	mRNA 3’-UTR binding	4.54E-15	ELAVL2/ELAVL3/ELAVL4/APOBEC1/IGF2BP1/IGF2BP2/IGF2BP3/DND1/RC3H1/ZFP36L1/RBM24/HNRNPA0/CPEB1/CPEB3/MEX3D/YBX3/RBM4	17
MF	GO:0004519	endonuclease activity	6.63E-15	ZC3H12C/RPP25/RNASEK/PLD6/PIWIL1/POP7/RNASE2/RNASE3/RNASE4/RNASE7/RNASE8/ANG/POP4/RNASE11/ENDOU/POP1/RNASEH2A/SMG6/RNASET2/EXO1	20
MF	GO:0045182	translation regulator activity	1.47E-13	SAMD4A/BOLL/DAZ1/RPS27L/IGF2BP1/IGF2BP2/IGF2BP3/EIF2AK2/EIF2AK4/CELF4/CPEB1/CPEB3/NANOS1/JAKMIP1	14
MF	GO:0140101	catalytic activity, acting on a tRNA	4.31E-12	METTL2A/TRMT5/RPP25/DARS2/TRMT10C/POP7/SARS2/POP4/PUS10/AARSD1/AARS2/PUS3/TRDMT1/FARSB/POP1/BCDIN3D/MARS2	17
MF	GO:0003729	mRNA binding	5.76E-12	ELAVL2/ELAVL3/ELAVL4/APOBEC1/EIF4A1/IGF2BP1/IGF2BP2/IGF2BP3/DND1/NXF3/PIWIL1/ACO1/LARP6/RC3H1/ZFP36L1/RBM24/RBPMS2/HNRNPA0/DHX33/CPEB1/CPEB3/MEX3D/RBM15/PPIE/YBX3/ESRP1/ESRP2/NANOS2/NOVA1/SRRM4/LIN28A/RBM4/JAKMIP1	33
MF	GO:0003725	double-stranded RNA binding	6.98E-12	OASL/OAS2/OAS3/MBNL1/EIF4A1/DHX58/SIDT2/RC3H1/EIF2AK2/YRDC/TLR7/TLR3/DHX33/DDX60	14
MF	GO:0004521	endoribonuclease activity	9.82E-12	RPP25/RNASEK/PIWIL1/POP7/RNASE4/RNASE8/POP4/ENDOU/POP1/RNASEH2A/SMG6/RNASET2/EXO1	13

**Table 2:** Pathway analysis by KEGG.

ID	Description	pvalue	geneID	Count
hsa03015	mRNA surveillance pathway	1.31E-14	MSI1/MSI2/GSPT2/CLP1/NXF2B/NXF2/NXF3/SRRM1/CSTF2/CSTF2T/PCF11/PABPC3/PABPC1L2A/PABPC1L2B/SMG6/PAPOLB/RNGTT	17
hsa03013	RNA transport	1.32E-14	RPP25/EIF4A1/NXF2B/NXF2/NXF3/POP7/SNUPN/GEMIN6/EIF3C/EIF3CL/POP4/SRRM1/EIF1AY/EEF1A2/SMN1/SMN2/PABPC3/PABPC1L2A/PABPC1L2B/POP1/EIF4E1B/EIF4E	22
hsa03008	Ribosome biogenesis in eukaryotes	7.89E-10	REXO2/RBM28/RPP25/RCL1/PWP2/EMG1/NXF2B/NXF2/NXF3/POP7/BMS1/POP4/UTP14C/POP1	14
hsa03018	RNA degradation	2.28E-07	PNLDC1/EXOSC7/EXOSC6/LSM3/CNOT2/PABPC3/PABPC1L2A/PABPC1L2B/CNOT6/WDR61	10
hsa03010	Ribosome	3.70E-06	RPS27L/RPL10L/RPS29/RPL17/MRPS18C/MRPL22/RPL3L/MRPL14/RPL22L1/RPL36A/MRPS6/MRPL23	12
hsa00970	Aminoacyl-tRNA biosynthesis	5.44E-06	DARS2/PSTK/SARS2/PARS2/AARS2/CARS2/FARSB/MARS2	8
hsa03040	Spliceosome	7.19E-05	CDC5L/LSM3/PRPF6/U2AF1/U2AF1L4/RBMXL3/HNRNPA1L2/HNRNPCL1/PPIE/PRPF40B	10
hsa03020	RNA polymerase	0.001088	POLR2J2/POLR2J3/POLR2D/POLR2F	4
hsa05164	Influenza A	0.003824	OAS2/OAS3/NXF2B/NXF2/NXF3/EIF2AK2/TLR7/TLR3	8

### PPI network building and subnet detection

**Fig 4:**
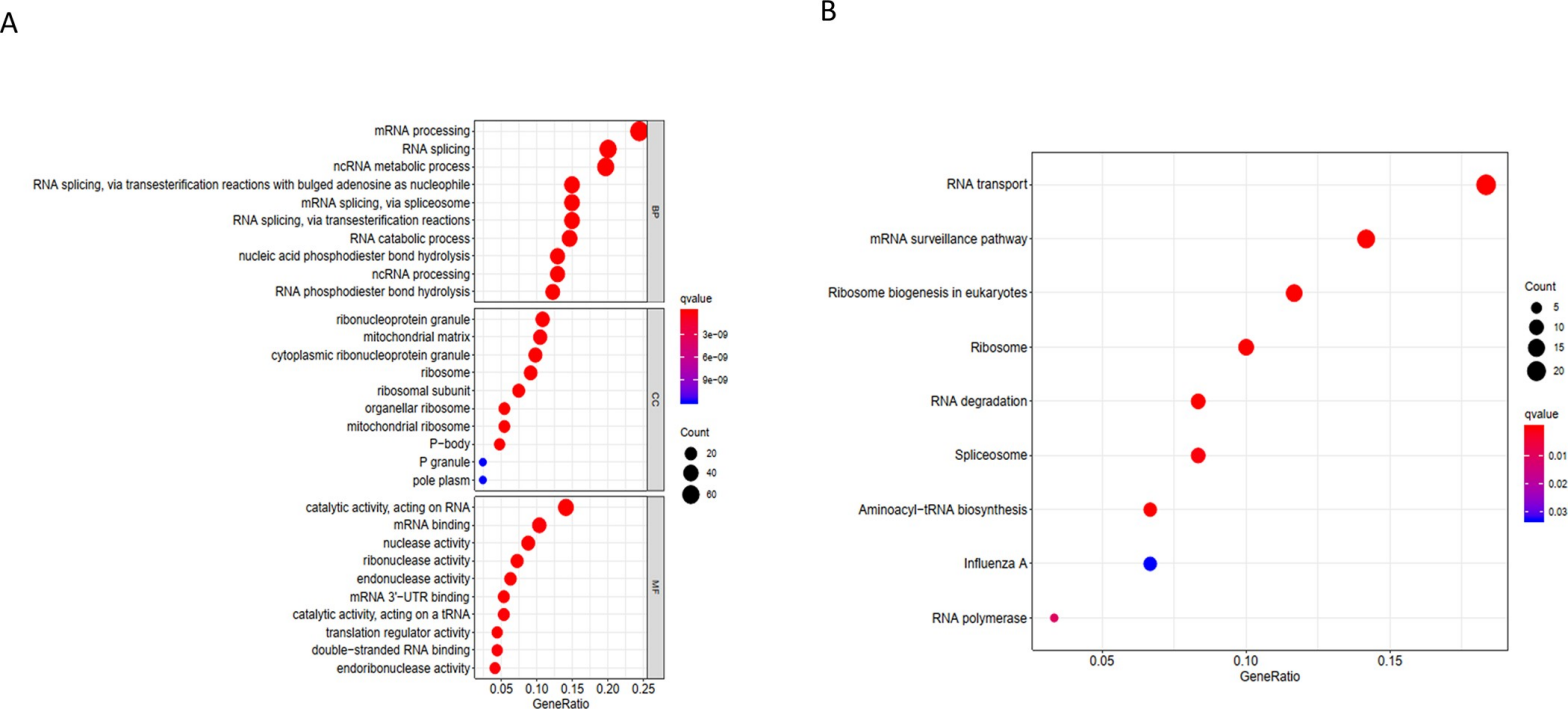
Functional characteristics of RBPs signature NB. (A)Gene Ontology analysis of biological processes was performed to analyze signature positively related gene. (B) The pathway in KEGG analysis (20).

To further study the function of differentially expressed RBPs and their role in the development of NB, we used Cytoscape software to create a PPI network that contained 311 nodes and 1766 edges. The co-expression network was analyzed using MCODE to recognize potential key modules (Fig 4). The RBPs in subgroup 1 were mainly enriched in ribosome biogenesis in the eukaryote pathway, ribosome biogenesis, rRNA processing, ncRNA processing, maturation of SSU-rRNA, ribosomal small subunit biogenesis, rRNA metabolic process, maturation of SSU-rRNA from tricistronic rRNA transcript (SSU-rRNA, 5.8S rRNA, LSU-rRNA), and in ribosomal large subunit biogenesis.

### Selection of prognosis-related RBP

**Fig 5:**
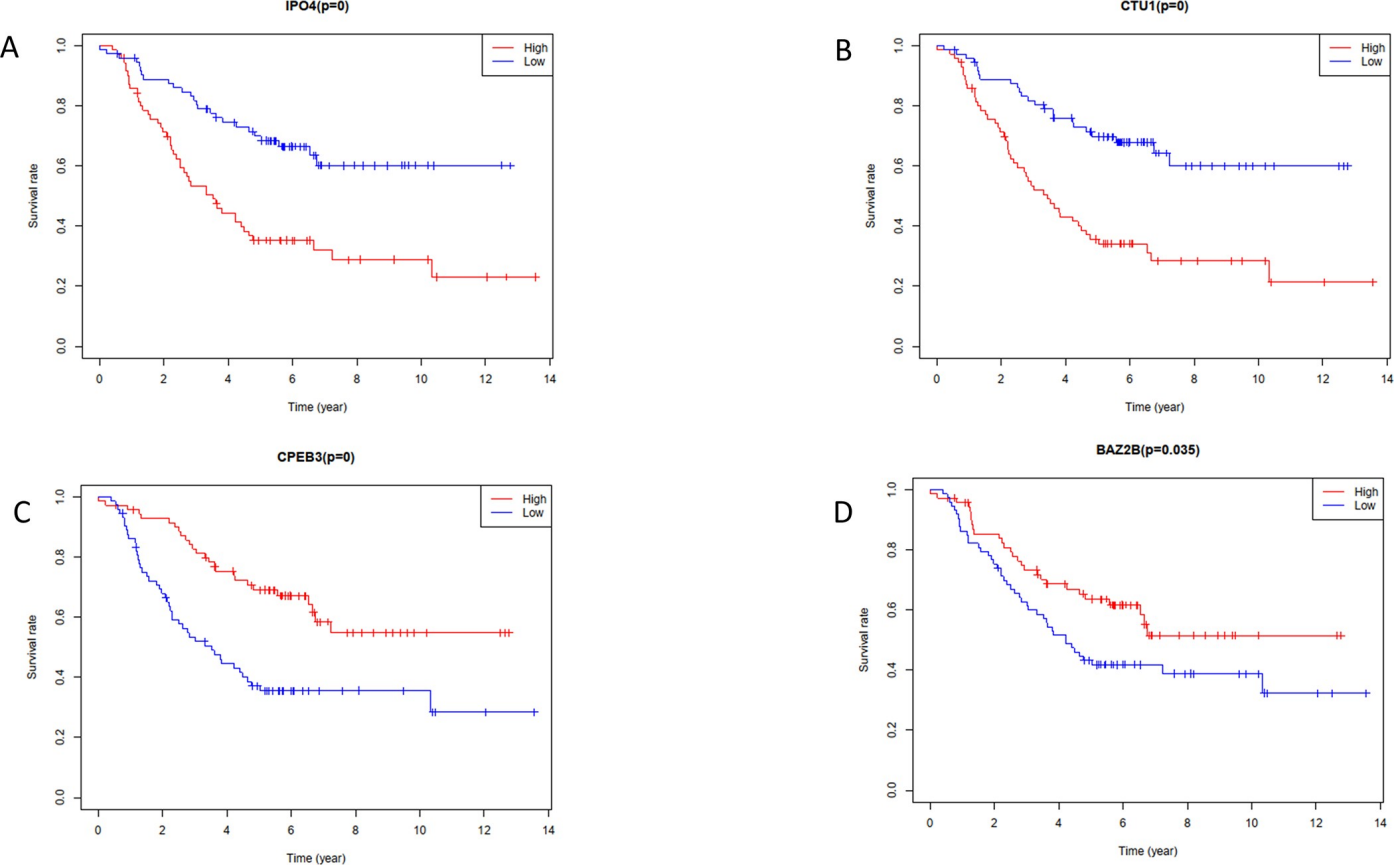
Survival analysis RBPs. (A) IPO4, (B) CTU1, ©CPEB3, (D) BAZ2B.

**Fig 6:**
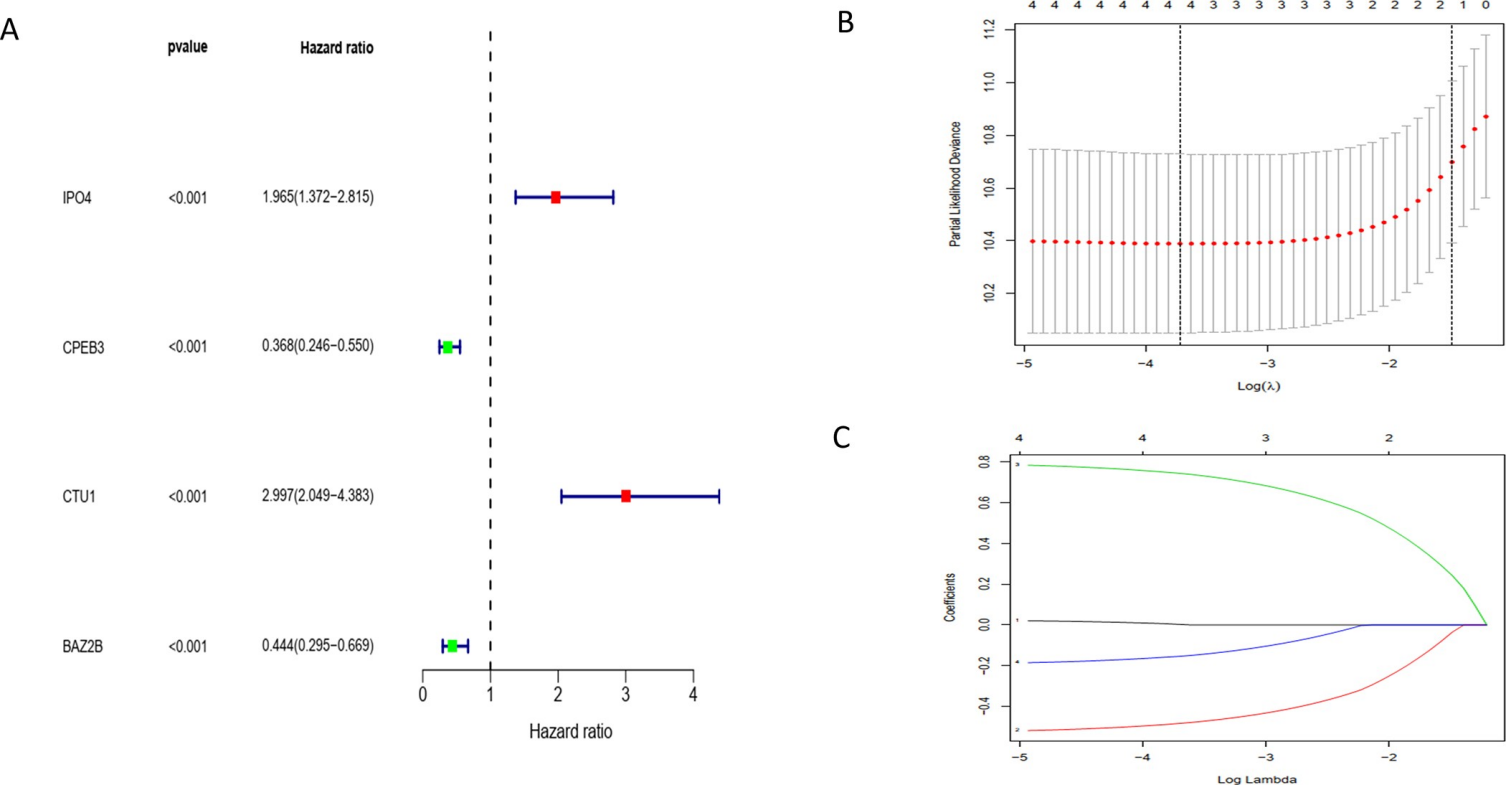
(A) Four survival prognosis-related genes identified by univariate cox analysis。 (B,C)The result of Lasso analysis.

Difference analysis identified a total of 348 key RBPs. To identify the prognostic significance of these RBPs and their effects on clinical outcome and survival, we performed univariate Cox regression analysis and obtained four candidate RBPs related to prognosis (Fig 5). Subsequently, using Lasso regression, the prognostic risk score including multi-factor Cox regression values was constructed (Fig 6, Table 3).

**Table 3:** Two hub RBPs identified from Cox regression analysis from TARGET dataset.

id	coef	HR	HR.95L	HR.95H	pvalue
CPEB3	-0.60901	0.543889	0.34522	0.856888	0.008642
CTU1	0.851637	2.34348	1.528648	3.59265	9.35E-05

### Prognosis-related RBPs model building and analysis

CPEB3 and CTU1 were identified as the key prognostic genes using the multivariate Cox regression analysis. We used these two hub genes to construct the predictive model. The risk score for each child was calculated based on the following formula:

Riskscore=(‐0.60901*expCPEB3)+(0.851637*expCTU1) (2)

**Fig 7:**
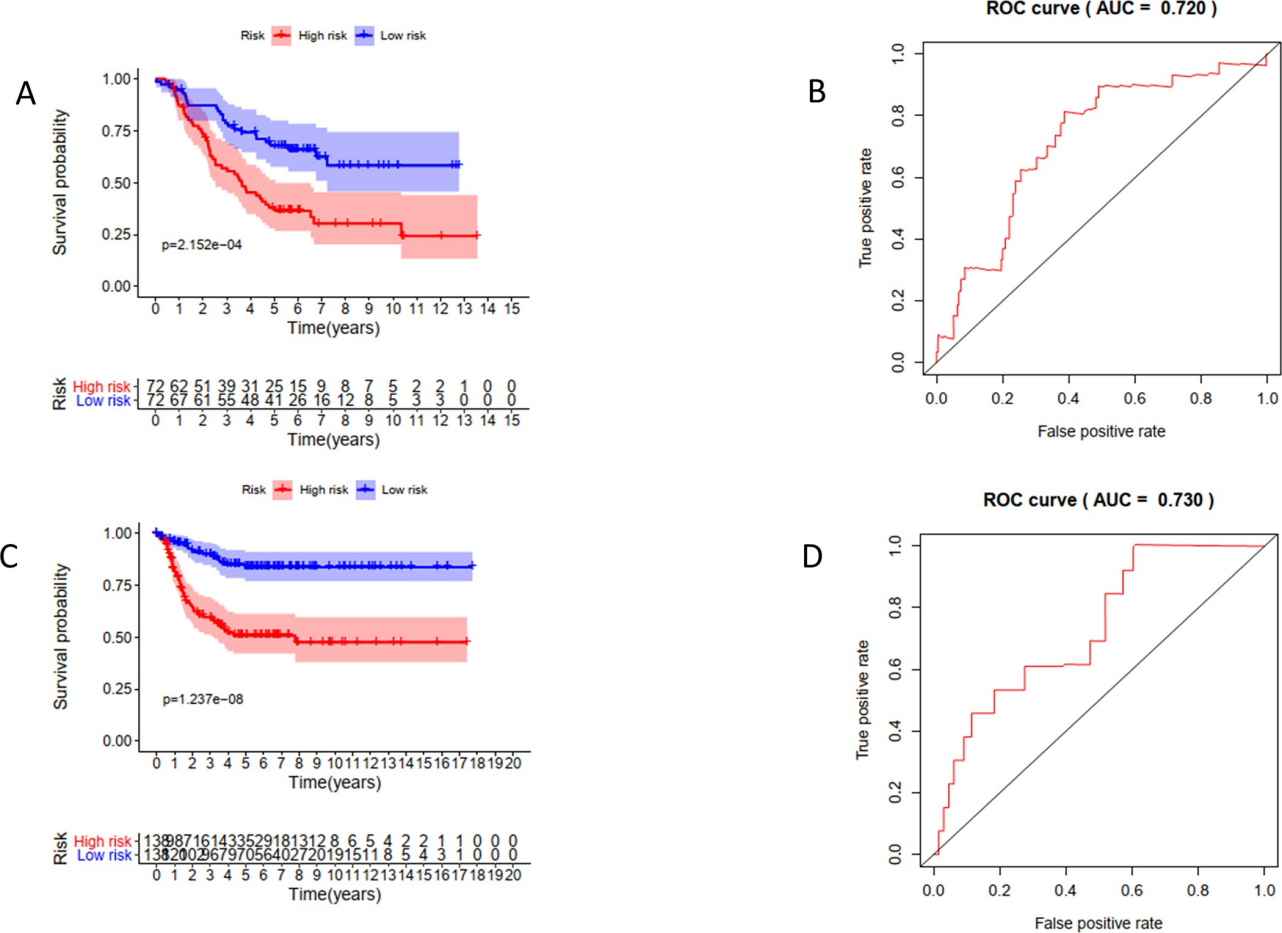
Survival analysis and prognostic risk assessment of 2-hub genes model for NB patients. (A,B) TARGET cohort, (C,D) GSE85047 cohort.

**Fig 8:**
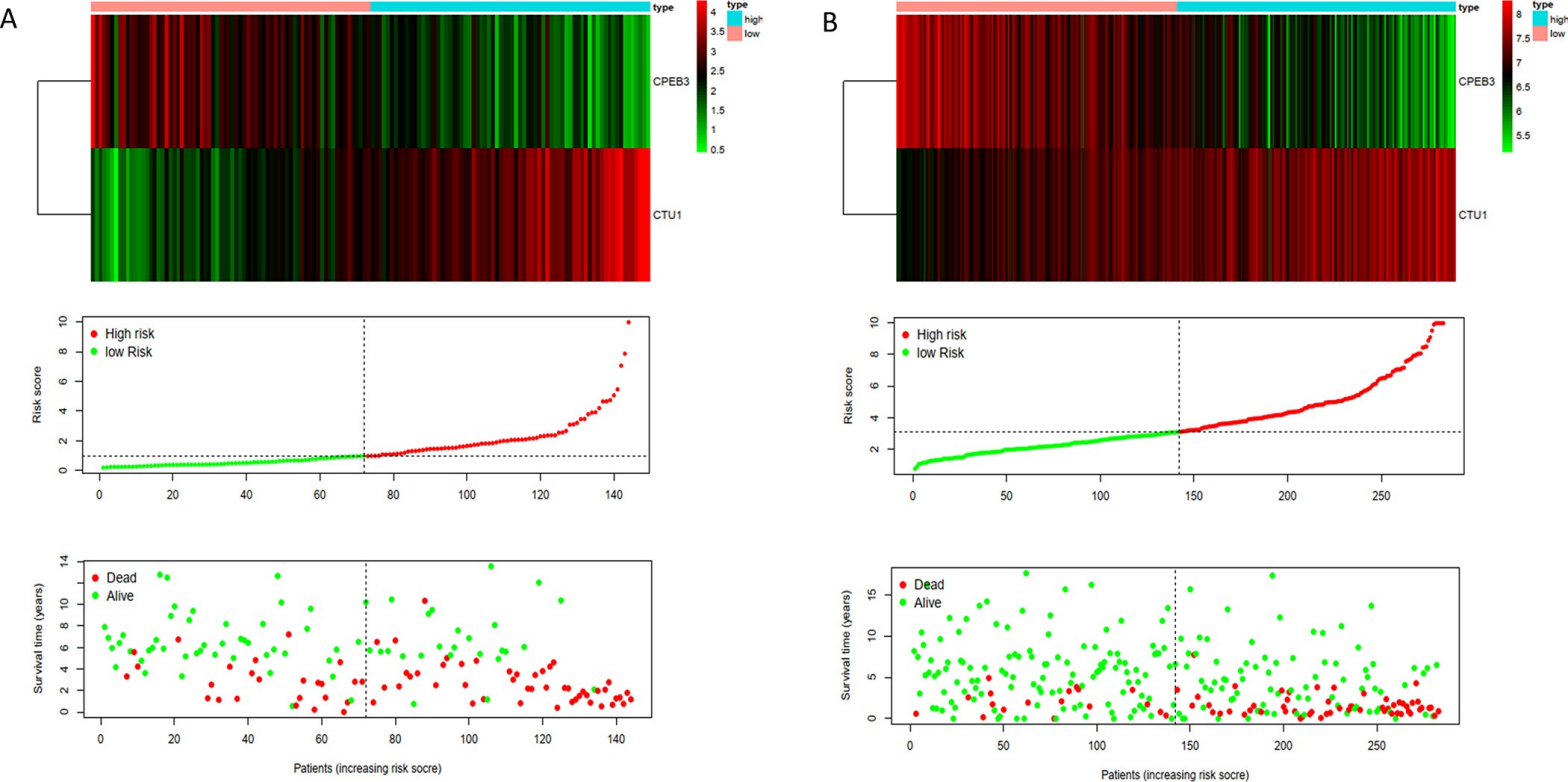
Risk score analysis of 2-gene prognostic model. (A) TARGET cohort. (B) GSE85047 cohort.

Then, based on the median value of the individual risk scores, 144 NB patients were stratified into two groups: the low-risk and high-risk groups. The results showed that compared with patients in the low-risk group, patients in the high-risk group had significantly poorer survival (P = 2.152e-04). The value of the area under the curve (AUC) in the TARGET model was 0.720 (Figs 7A, 7B, and 8A).

### Validation of hub RBPs

To evaluate the prognostic value of the RBP prediction model, we used the GSE85047 patient cohort to verify the relationship between risk score and survival. In the GSE85047 cohort, groups were also grouped based on the median value of risk score in the TARGET model. The survival of patients with high-risk scores was poorer than for patients having lower risk scores (P = 0.1237e-08), and the AUC was 0.730 (Figs 7C, 7D, and 8B).

### The RBP risk score was an independent prognostic factor

**Fig 9:**
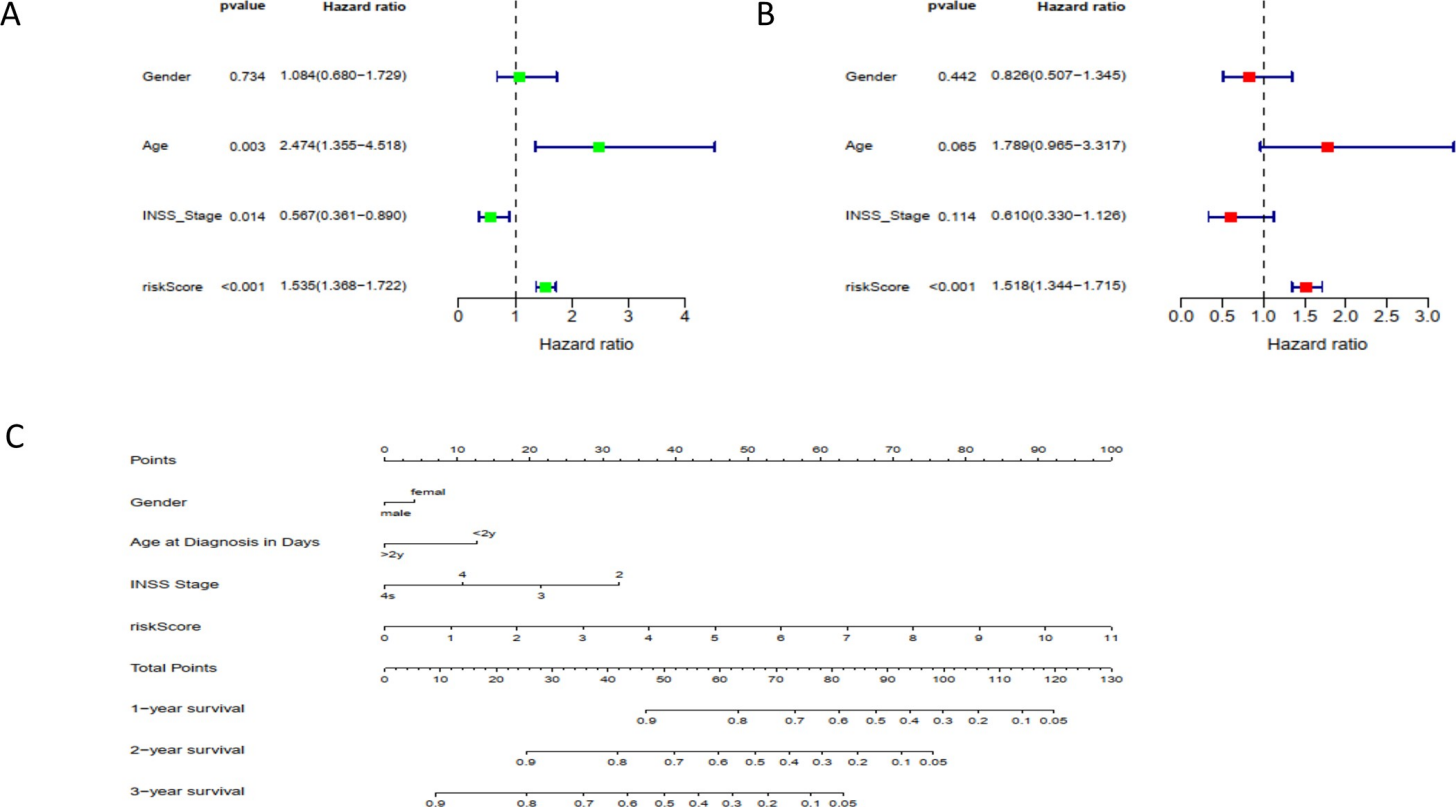
The nomogram can predict the prognosis probability in NB. A. Univariate Cox regression analysis. Forest plot of associations between risk factors and the survival of NB. B. Multiple Cox regression analysis. The RBPs gene signature is an independent predictor of NB. C. Nomogram of the NB cohort used to predict the OS.

We assessed the prognostic value of risk scores of RBPs. For the NB TARGET cohort, the risk scores on univariate analysis were significantly correlated with overall survival (OS) (HR = 1.535, 95% CI = 1.368–1.722, P = 2.69E-13) (Fig 9A). The multivariate analysis showed that the risk score was an independent prognostic indicator (HR = 1.518, 95% CI = 1.344–1.715, P = 1.91E-11) (Fig 9B). We constructed a nomogram that integrated multiple risk factors to quantify individual risks to be used in the clinical setting to predict the OS probability at 1, 2, and 3 years (Fig 9C).

### Constructing the lncRNA-miRNA-mRNA network of key RBPs

**Fig 10:**
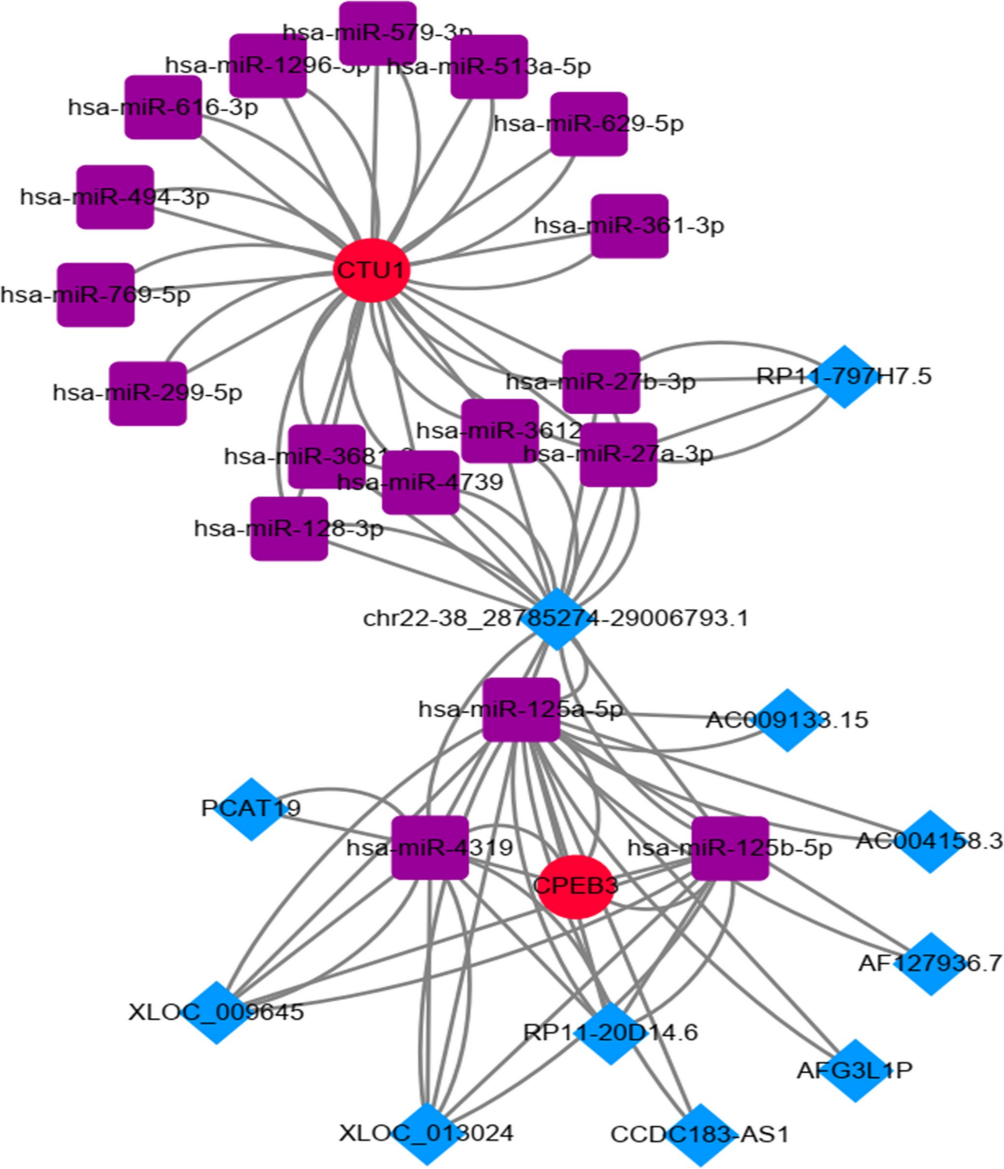
The lncRNA-miRNA-mRNA network of CPEB3 and CTU1.

Using online databases, we constructed the lncRNA-miRNA-mRNA network of key RBPs (Fig 10).

## Discussion

The prognosis of different NB patients varies greatly, given the extensive tumor heterogeneity of NB. For low-risk NB patients (most commonly infants), simple observation or surgical treatment can often achieve good results; but for high-risk NB patients, even if a variety of intensive treatment options are combined [25], the prognosis is still not ideal. The true cause of NB is still unclear. In recent years, with the emergence of immunotherapy and new drugs, the survival of patients in the high-risk group has improved to a certain extent [26].

RBPs have always played a role in the life of RNA. It is not an exaggeration to say that without RBP, RNA cannot achieve anything. The main role of RBPs is to mediate RNA maturation, transport, localization, and translation; one RBP may have multiple target RNAs, and defect in expression may cause multiple diseases. Recently, the importance of RBP in tumor occurrence, development, and metastasis has increasingly been revealed [27].

In our study, we identified 348 RBPs based on NB datasets from the TARGET NB dataset. We systematically analyzed the related biological functions and built RBP PPI networks and the relative subnets. In addition, we performed univariate Cox regression analysis, survival analysis, Lasso regression analysis, and multivariate Cox regression analysis of differential RBPs to further investigate their biological function and prognostic value.

The GO enrichment and KEGG pathway analysis of these differentially expressed RBPs indicated that RBP were used in mRNA monitoring pathways, RNA transport, ribosomal biogenesis in eukaryotes, RNA degradation, ribosomes, aminoacyl-tRNA biosynthesis, and spliceosomes. RBPs were significantly enriched in the RNA polymerase pathway, and play a critical role in mRNA processing, RNA splicing, ncRNA metabolism, RNA phosphodiester bond hydrolysis, catalytic activity, and act on RNA ribonuclease and nuclease activity. The PPI network revealed the relationship between RBPs. At present, with the development of computational biology, it is possible to predict the complex regulatory relationship between proteins, which provides a powerful tool for further research [28]. At present, many studies have described the role of RBPs role in metabolism and disease. RBPs plays a dual and opposite role in tumorigenesis, regulating the proliferation of early tumor cells and promoting tumor progression and metastasis in advanced cancer. According to these reports, abnormal expression of multiple RBPs had been found in many malignant tumors [9, 29, 30]. However, the impact of RBP on the occurrence and development of cancer is still poorly understood.

In our study, two RBPs were identified as hub RBPs related to NB prognosis: *CPEB3* and *CTU1*. *CPEB3*, also known as the Cytoplasmic Polyadenylation Element Binding Protein is an RBP that shuttles through the cytoplasm. It mainly exists in the cytoplasm and can inhibit the translation of target RNA. GO analysis showed that *CPEB3* was associated with mRNA processing, RNA catabolic processes, mRNA 3’-UTR binding, translation regulator activity, and mRNA binding. *CPEB3* can disrupt the crosstalk between cancer cells and tumor-associated macrophages through the IL-6R/STAT3 signaling pathway, and thereby inhibit epithelial-mesenchymal transition. Studies have found that *CPEB3* is related to the tumorigenesis and development of glioma [31], high-grade serous ovarian cancer [32], colorectal cancer [33], hepatocellular carcinoma [34], and cervical cancer [35]. *CPEB3* is expressed in both the brain and heart and is involved in the regulation of synaptic plasticity by down-regulating the expression of several plasticity-related proteins (PRPs), such as N-methyl-D-aspartate receptor (NMDAR) and postsynaptic density protein 95 (PSD95). Down-regulation of CPEB3 expression can affect NMDAR activated CaMKII α, which overcomes the inhibition of synaptic transmission under stress [36, 37]. In addition, *CPEB3* knockout mice exhibited hippocampus-dependent neurological dysfunction, including acquisition and extinction of long-term spatial memory and short-term fear memory.

In nerve cells, activation of NMDAR can increase the expression of *CPEB3* in the nucleus and redistributes its content in nucleus and cytoplasm [38]. *CPEB3* has low expression in colon cancer and HPV-positive cervical cancer, which indicates it may be involved in tumorigenesis as a tumor suppressor, but the specific mechanism still needs further study [39]. CPEB protein family members contain multiple miRNA binding sites, and can be regulated by a variety of miRNAs [40]. After up regulating the expression of mir-107 in hepatoma cell lines Huh7 and HepG2, the mir-107 bound to the 3‘-UTR region of the *CPEB3* transcript, and resulted in an increased expression of EGFR and phosphorylated Akt and decreased expression of p21. Mir-107 and CPEB3 interaction plays an important role in the occurrence and development of hepatocellular carcinoma by regulating the EGFR signaling pathway [34].

*CTU1*, or cytoplasmic sulfurylase subunit 1, is a protein-coding gene. Diseases associated with CTU1 include spinal cord septal membrane tumors and spinal gliomas. The related pathways include gene expression and tRNA processing, and its function is related to tRNA binding and nucleotide transferase activity. GO analysis shows *CTU1* was associated with ncRNA metabolic process and ncRNA processing. *CTU1* promotes cancer resistance to targeted therapy [41], its expression is associated with higher rates of morbidity and mortality in spinal cord gliomas [42], and up-regulation of *CTU1* is involved in human breast cancer metastasis.

In our study, based on the two hubs RBPs identified in the TARGET cohort training set, multi-step Cox regression analysis produced a risk score model that could predict the prognosis of NB. In the NB TARGET and GSE85047 cohorts, the survival outcomes of the high- and low-risk subgroups were significantly different. The ROC values of the risk score model of the training set and validation set were 0.72 and 0.73, respectively, indicating that the 2-gene marker prognostic model applied to evaluate the prognosis of NB patients has a certain value. Both genes were differentially expressed in NB and correlated with survival, which strongly suggests that these two genes are potential tumor-related genes and require further study. In the future, we intend to establish a real-life cohort of NB patients to confirm the validity of these genes. However, the molecular mechanisms involving these two RBPs are unknown, and further study of their underlying function may be valuable.

In recent years, more and more studies have confirmed that long non-coding RNA (lncRNA) and micro RNA (miRNA) and their interactions play an important role in the diagnostic biomarkers and therapeutic targets of various diseases. Some theoretical methods have played a role in predicting potential lncRNA-miRNA associations. With the development of bioinformatics, some new prediction methods, such as the lncRNA–miRNA interactions prediction by logistic matrix factorization with neighborhood regularized (LMFNRLMI), enable us to study lncRNA-miRNA interactions more accurately, that is, to predict lncRNA-miRNA interactions [43].

In summary, we systematically studied the function and prognostic value of RBPs differently expressed in NB. These RBPs may be associated with the occurrence, development, invasion, and metastasis of NB. The establishment of a prognostic model of NB based on two RBP coding genes is conducive to clinical application. Our results contribute to a better understanding of the pathogenesis of NB and the development of new therapeutic and prognostic molecular markers. Although our gene signature and nomogram showed excellent performance in the training set and validation set, both inevitably had some limitations. First, although our risk score performed well in predicting the survival rate of NB patients, it lacks confirmation from large-scale prospective trials. Secondly, the validation data derived only from the GSE 85047 dataset; thus, the predictive value of our model requires further verification. Third, the molecular mechanisms involving *CPEB3* and *CTU1* have not been verified in NB cells. Thus, our follow-up studies will verify the conclusions reached in this study from the aspect of their clinical application and molecular mechanisms.

## Supporting information

S1 Table Differently expressed RBPs in NB patients. (XLS)

## References

[1] Kamihara J, Bourdeaut F, Foulkes WD, Molenaar JJ, Mossé YP, Nakagawara A, et al. Retinoblastoma and Neuroblastoma Predisposition and Surveillance. Clin Cancer Res. 2017;23(13). doi: 10.1158/1078-0432.CCR-17-0652 . 28674118 PMC7266051

[2] Matthay KK, Maris JM, Schleiermacher G, Nakagawara A, Mackall CL, Diller L, et al. Neuroblastoma. Nat Rev Dis Primers. 2016;2:16078. doi: 10.1038/nrdp.2016.78 . 27830764

[3] Pinto NR, Applebaum MA, Volchenboum SL, Matthay KK, London WB, Ambros PF, et al. Advances in Risk Classification and Treatment Strategies for Neuroblastoma. J Clin Oncol. 2015;33(27):3008–17. doi: 10.1200/JCO.2014.59.4648 . 26304901 PMC4567703

[4] Oldridge DA, Truong B, Russ D, DuBois SG, Vaksman Z, Mosse YP, et al. Differences in Genomic Profiles and Outcomes Between Thoracic and Adrenal Neuroblastoma. J Natl Cancer Inst. 2019;111(11):1192–201. doi: 10.1093/jnci/djz027 . 30793172 PMC6855946

[5] Jones DTW, Banito A, Grünewald TGP, Haber M, Jäger N, Kool M, et al. Molecular characteristics and therapeutic vulnerabilities across paediatric solid tumours. Nat Rev Cancer. 2019;19(8):420–38. doi: 10.1038/s41568-019-0169-x . 31300807

[6] Hentze MW, Castello A, Schwarzl T, Preiss T. A brave new world of RNA-binding proteins. Nat Rev Mol Cell Biol. 2018;19(5):327–41. doi: 10.1038/nrm.2017.130 . 29339797

[7] Ramanathan M, Majzoub K, Rao DS, Neela PH, Zarnegar BJ, Mondal S, et al. RNA-protein interaction detection in living cells. Nat Methods. 2018;15(3):207–12. doi: 10.1038/nmeth.4601 . 29400715 PMC5886736

[8] Gerstberger S, Hafner M, Tuschl T. A census of human RNA-binding proteins. Nat Rev Genet. 2014;15(12):829–45. doi: 10.1038/nrg3813 . 25365966 PMC11148870

[9] Pereira B, Billaud M, Almeida R. RNA-Binding Proteins in Cancer: Old Players and New Actors. Trends Cancer. 2017;3(7):506–28. doi: 10.1016/j.trecan.2017.05.003 . 28718405

[10] Dominguez D, Freese P, Alexis MS, Su A, Hochman M, Palden T, et al. Sequence, Structure, and Context Preferences of Human RNA Binding Proteins. Mol Cell. 2018;70(5). doi: 10.1016/j.molcel.2018.05.001 . 29883606 PMC6062212

[11] Chu C, Zhang QC, da Rocha ST, Flynn RA, Bharadwaj M, Calabrese JM, et al. Systematic discovery of Xist RNA binding proteins. Cell. 2015;161(2):404–16. doi: 10.1016/j.cell.2015.03.025 . 25843628 PMC4425988

[12] Lee FCY, Ule J. Advances in CLIP Technologies for Studies of Protein-RNA Interactions. Mol Cell. 2018;69(3):354–69. doi: 10.1016/j.molcel.2018.01.005 . 29395060

[13] Gupta SK, Garg A, Bär C, Chatterjee S, Foinquinos A, Milting H, et al. Quaking Inhibits Doxorubicin-Mediated Cardiotoxicity Through Regulation of Cardiac Circular RNA Expression. Circ Res. 2018;122(2):246–54. doi: 10.1161/CIRCRESAHA.117.311335 . 29133306 PMC5771684

[14] Zu T, Cleary JD, Liu Y, Bañez-Coronel M, Bubenik JL, Ayhan F, et al. RAN Translation Regulated by Muscleblind Proteins in Myotonic Dystrophy Type 2. Neuron. 2017;95(6). doi: 10.1016/j.neuron.2017.08.039 . 28910618 PMC5951173

[15] Bennett CL, Dastidar SG, Ling S-C, Malik B, Ashe T, Wadhwa M, et al. Senataxin mutations elicit motor neuron degeneration phenotypes and yield TDP-43 mislocalization in ALS4 mice and human patients. Acta Neuropathol. 2018;136(3):425–43. doi: 10.1007/s00401-018-1852-9 . 29725819 PMC6098723

[16] Chatterji P, Rustgi AK. RNA Binding Proteins in Intestinal Epithelial Biology and Colorectal Cancer. Trends Mol Med. 2018;24(5):490–506. doi: 10.1016/j.molmed.2018.03.008 . 29627433 PMC5927824

[17] Zhao Q, Yu H, Ming Z, Hu H, Ren G, Liu H. The Bipartite Network Projection-Recommended Algorithm for Predicting Long Non-coding RNA-Protein Interactions. Mol Ther Nucleic Acids. 2018;13:464–471. doi: 10.1016/j.omtn.2018.09.020 . 30388620 PMC6205413

[18] Zhao Q, Zhang Y, Hu H, Ren G, Zhang W, Liu H. IRWNRLPI: Integrating Random Walk and Neighborhood Regularized Logistic Matrix Factorization for lncRNA-Protein Interaction Prediction. Front Genet. 2018;9:239. doi: 10.3389/fgene.2018.00239 . 30023002 PMC6040094

[19] Hu H, Zhang L, Ai H, Zhang H, Fan Y, Zhao Q, et al. HLPI-Ensemble: Prediction of human lncRNA-protein interactions based on ensemble strategy. RNA Biol. 2018;15(6):797–806. doi: 10.1080/15476286.2018.1457935 . 29583068 PMC6152435

[20] Yu G, Wang L-G, Han Y, He Q-Y. clusterProfiler: an R package for comparing biological themes among gene clusters. OMICS. 2012;16(5):284–7. doi: 10.1089/omi.2011.0118 . 22455463 PMC3339379

[21] Szklarczyk D, Gable AL, Lyon D, Junge A, Wyder S, Huerta-Cepas J, et al. STRING v11: protein-protein association networks with increased coverage, supporting functional discovery in genome-wide experimental datasets. Nucleic Acids Res. 2019;47(D1):D607–D13. doi: 10.1093/nar/gky1131 . 30476243 PMC6323986

[22] Bader GD, Hogue CWV. An automated method for finding molecular complexes in large protein interaction networks. BMC Bioinformatics. 2003;4:2. doi: 10.1186/1471-2105-4-2 . 12525261 PMC149346

[23] Garcia DM, Baek D, Shin C, Bell GW, Grimson A, Bartel DP. Weak seed-pairing stability and high target-site abundance decrease the proficiency of lsy-6 and other microRNAs. Nat Struct Mol Biol. 2011;18(10):1139–46. doi: 10.1038/nsmb.2115 . 21909094 PMC3190056

[24] Kanehisa M, Goto S. KEGG: kyoto encyclopedia of genes and genomes. Nucleic Acids Res. 2000;28(1):27–30. Epub 1999/12/11. doi: 10.1093/nar/28.1.27 ; PubMed Central PMCID: PMC102409. 10592173 PMC102409

[25] Ackermann S, Cartolano M, Hero B, Welte A, Kahlert Y, Roderwieser A, et al. A mechanistic classification of clinical phenotypes in neuroblastoma. Science (New York, NY). 2018;362(6419):1165–70. doi: 10.1126/science.aat6768 . 30523111 PMC7875194

[26] Fletcher JI, Ziegler DS, Trahair TN, Marshall GM, Haber M, Norris MD. Too many targets, not enough patients: rethinking neuroblastoma clinical trials. Nat Rev Cancer. 2018;18(6):389–400. doi: 10.1038/s41568-018-0003-x . 29632319

[27] Wang E, Lu SX, Pastore A, Chen X, Imig J, Chun-Wei Lee S, et al. Targeting an RNA-Binding Protein Network in Acute Myeloid Leukemia. Cancer Cell. 2019;35(3). doi: 10.1016/j.ccell.2019.01.010 . 30799057 PMC6424627

[28] Zhang L, Liu T, Chen H, Zhao Q, Liu H. Predicting lncRNA-miRNA interactions based on interactome network and graphlet interaction. Genomics. 2021;113(3):874–80. doi: 10.1016/j.ygeno.2021.02.002 . 33588070

[29] Lujan DA, Ochoa JL, Hartley RS. Cold-inducible RNA binding protein in cancer and inflammation. Wiley Interdiscip Rev RNA. 2018;9(2). doi: 10.1002/wrna.1462 . 29322631 PMC5886743

[30] Degrauwe N, Suvà M-L, Janiszewska M, Riggi N, Stamenkovic I. IMPs: an RNA-binding protein family that provides a link between stem cell maintenance in normal development and cancer. Genes Dev. 2016;30(22):2459–74. doi: 10.1101/gad.287540.116 . 27940961 PMC5159662

[31] Chen Y, Bao C, Zhang X, Lin X, Huang H, Wang Z. Long non-coding RNA HCG11 modulates glioma progression through cooperating with miR-496/CPEB3 axis. Cell Prolif. 2019;52(5):e12615. doi: 10.1111/cpr.12615 . 31310044 PMC6797506

[32] Liu F, Zhang G, Lv S, Wen X, Liu P. miRNA-301b-3p accelerates migration and invasion of high-grade ovarian serous tumor via targeting CPEB3/EGFR axis. J Cell Biochem. 2019;120(8):12618–27. doi: 10.1002/jcb.28528 . 30834603

[33] Zhong Q, Fang Y, Lai Q, Wang S, He C, Li A, et al. CPEB3 inhibits epithelial-mesenchymal transition by disrupting the crosstalk between colorectal cancer cells and tumor-associated macrophages via IL-6R/STAT3 signaling. J Exp Clin Cancer Res. 2020;39(1):132. doi: 10.1186/s13046-020-01637-4 . 32653013 PMC7353816

[34] Zou C-D, Zhao W-M, Wang X-N, Li Q, Huang H, Cheng W-P, et al. MicroRNA-107: a novel promoter of tumor progression that targets the CPEB3/EGFR axis in human hepatocellular carcinoma. Oncotarget. 2016;7(1):266–78. doi: 10.18632/oncotarget.5689 . 26497556 PMC4807997

[35] Zhang Y, Yu R, Li L. LINC00641 hinders the progression of cervical cancer by targeting miR-378a-3p/CPEB3. J Gene Med. 2020:e3212. doi: 10.1002/jgm.3212 . 32367630

[36] Giangarrà V, Igea A, Castellazzi CL, Bava F-A, Mendez R. Global Analysis of CPEBs Reveals Sequential and Non-Redundant Functions in Mitotic Cell Cycle. PLoS One. 2015;10(9):e0138794. doi: 10.1371/journal.pone.0138794 . 26398195 PMC4580432

[37] Kozlov E, Shidlovskii YV, Gilmutdinov R, Schedl P, Zhukova M. The role of CPEB family proteins in the nervous system function in the norm and pathology. Cell Biosci. 2021;11(1):64. doi: 10.1186/s13578-021-00577-6 . 33789753 PMC8011179

[38] Fioriti L, Myers C, Huang Y-Y, Li X, Stephan JS, Trifilieff P, et al. The Persistence of Hippocampal-Based Memory Requires Protein Synthesis Mediated by the Prion-like Protein CPEB3. Neuron. 2015;86(6):1433–48. doi: 10.1016/j.neuron.2015.05.021 . 26074003

[39] D’Ambrogio A, Nagaoka K, Richter JD. Translational control of cell growth and malignancy by the CPEBs. Nat Rev Cancer. 2013;13(4):283–90. doi: 10.1038/nrc3485 . 23446545

[40] Fernández-Miranda G, Méndez R. The CPEB-family of proteins, translational control in senescence and cancer. Ageing Res Rev. 2012;11(4):460–72. doi: 10.1016/j.arr.2012.03.004 . 22542725

[41] Rapino F, Delaunay S, Rambow F, Zhou Z, Tharun L, De Tullio P, et al. Codon-specific translation reprogramming promotes resistance to targeted therapy. Nature. 2018;558(7711):605–9. doi: 10.1038/s41586-018-0243-7 . 29925953

[42] Zhang M, Iyer RR, Azad TD, Wang Q, Garzon-Muvdi T, Wang J, et al. Genomic Landscape of Intramedullary Spinal Cord Gliomas. Sci Rep. 2019;9(1):18722. doi: 10.1038/s41598-019-54286-9 . 31822682 PMC6904446

[43] Liu H, Ren G, Chen H, Liu Q, Zhao Q. Predicting lncRNA-miRNA interactions based on logistic matrix factorization with neighborhood regularized. Knowledge-Based Systems. 2019;191.105261.

